# Oxymatrine Inhibits Twist-Mediated Renal Tubulointerstitial Fibrosis by Upregulating Id2 Expression

**DOI:** 10.3389/fphys.2020.00599

**Published:** 2020-06-19

**Authors:** Ying Xiao, Can Peng, Yawen Xiao, Dan Liang, Zhiping Yuan, Zhiyang Li, Mingjun Shi, Yuanyuan Wang, Fan Zhang, Bing Guo

**Affiliations:** ^1^Department of Pathophysiology, Guizhou Medical University, Guiyang, China; ^2^Guizhou Provincial Key Laboratory of Pathogenesis & Drug Research on Common Chronic Diseases, Guizhou Medical University, Guiyang, China; ^3^State Key Laboratory of Functions and Applications of Medicinal Plants, Guizhou Medical University, Guiyang, China; ^4^School Hospital, Guizhou Medical University, Guiyang, China

**Keywords:** oxymatrine, diabetes mellitus, renal tubulointerstitial fibrosis, inhibitor of differentiation 2, Twist

## Abstract

The final pathway for the development of diabetic nephropathy (DN) into chronic renal failure in DN is glomerulosclerosis and tubulointerstitial fibrosis. Renal tubular lesions can occur in the early stage of DN renal injury. Cumulative evidence shows that oxymatrine (OMT) has a variety of biological and pharmacological properties. In recent years, more attention has been paid on the preventive and therapeutic influence of OMT on organ fibrosis. In this experiment, db/db mice were intraperitoneally injected with OMT 120 mg/kg for 8 weeks, and NRK-52E cultured with 30 mmol/L glucose and 0.1 mg/mL OMT for 48-hour. We investigated the relationship between Id2 and Twist in NRK-52E cells and the effect of OMT on the expression of E-cadherin, α-SMA, Fibronectin, and Collagen-IV by Western blot, Real-time PCR, Immunofluorescence, cell transfection, Co-Immunoprecipitation, and Luciferase assays. OMT increased the expression of Id2 but decreased that of Twist under high glucose condition *in vitro* and *in vivo*. The promoted recovery of Id2 facilitated its binding to Twist and affected E-cadherin activity inhibiting EMT and the excessive proliferation and abnormal deposition of ECM. In brief, OMT promotes Id2 to reverse EMT and exert anti-fibrotic effect in diabetic renal tubular epithelial cells by binding Id2 to Twist and affecting its transcriptional activation of downstream target genes. Or findings provide a new experimental basis for delaying the progress and for treatment of diabetic renal fibrosis.

## Introduction

With the increase in the incidence of diabetes mellitus (DM) each year, diabetic nephropathy (DN), also known as diabetic kidney disease (DKD), has become one of its most serious complications. It is also one of the main causes of death in patients with DM. DN is also one of the main causes of end-stage renal disease globally and one of the main reasons for the decline of quality of life and increased mortality in patients with DM (Hakim and Pflueger, 2010). Therefore, it is of great clinical significance to clarify the occurrence and development mechanism of DN as soon as possible to provide an effective target for the prevention and treatment of DN and drug therapy of DN.

The main pathological features of DN are loss of normal nephron, proliferation of a large number of fibroblasts and myofibroblasts, excessive production and accumulation of extracellular matrix, thickening of the glomerular and tubular basement membrane, and renal tubulointerstitial fibrosis (Conserva et al., 2019; Li X. et al., 2019). Renal tubulointerstitial fibrosis is the most important renal interstitial lesion, which is almost the most common pathway and main pathological basis for the progression of various renal diseases to end-stage renal failure (Zhang et al., 2019). The process of renal tubulointerstitial fibrosis is epithelial–mesenchymal transition (EMT), which results in the loss of epithelioid properties and the acquisition of mesenchymal properties. The decreased expression of E-cadherin and increased the expression of α-SMA, accompanied by excessive proliferation and abnormal deposition of in extracellular matrix (ECM). The expression of Collagen-IV and Fibronectin was upregulated. Therefore, inhibiting or reversing the occurrence of EMT is of great significance in delaying chronic kidney diseases such as DN.

Oxymatrine (OMT) has many pharmacological effects, such as immune regulation, anti-arrhythmia, anti-inflammation, anti-tumor, anti-fibrosis (Ding et al., 2019; Halim et al., 2019; Jung et al., 2019; Wang et al., 2019b; Zhou et al., 2019; Lan et al., 2020). It has beneficial influence on myocardial fibrosis, hepatic fibrosis, pulmonary fibrosis and renal fibrosis in rats (Chen et al., 2008; Shen et al., 2011; Liu et al., 2012; Fu et al., 2016; Wang et al., 2016; Ozturk et al., 2017; Xu et al., 2017; Zhao et al., 2018). Our previous study showed that after OMT intervention of NRK-52E cells cultured with high glucose, the protein and mRNA levels of α-SMA and fibronectin decreased, while the protein and mRNA of E-cadherin increased, suggesting that OMT can inhibit the occurrence of EMT and improve the degree of renal fibrosis (Liu et al., 2016). Wang interfered with renal interstitial fibrosis induced by unilateral ureteral obstruction (UUO) in mice, which confirmed that OMT could significantly reduce the protein expression of Collagen-I and Fibronectin and prevent renal injury and renal interstitial fibrosis (Wang et al., 2016). However, the specific mechanism of OMT against renal tubulointerstitial fibrosis is yet to be elucidated.

The regulatory factors of fibrosis in a normal body are balanced with the anti-fibrotic ones. However, in the process of EMT progression, many fibrogenic regulators play a major role, and the expression of some anti-fibrotic regulators is reduced, such as inhibitors of differentiation (Yu et al., 2016; Cantelli et al., 2017; Higgins et al., 2017; Wang et al., 2018, Wang et al., 2019a; Zhao and Liu, 2020). Inhibitor of differentiation 2 (Id2), also known as Inhibitor of DNA binding, belongs to the helix-ring-helix family, which negatively regulates the activity of basic HLH (bHLH) transcription factors (Benezra et al., 1990; Zhou et al., 2018; Xiao et al., 2019; Jeyarajah et al., 2020; Xu et al., 2020). The binding region of bHLH regulates the expression of genes by binding to DNA in the form of dimers or heterodimers. However, Id2 protein lacks the basic region that binds to DNA and needs to bind to alkaline HLH to form a heterodimer, which then inhibits the regulation of alkaline HLH on gene and exerts negative effects on gene regulation (Norton, 2000; Roschger et al., 2018). Previous studies on diabetic rats established that the expression of Id2 in renal tubular epithelial cells decreased gradually with the progression of diabetes. The expression of E-cadherin and α-SMA decreased significantly, accompanied by the deposition of renal interstitial ECM. It is suggested that the EMT process of renal tubular epithelial cells may be related to the decrease of Id2 expression. Twist is a transcription factor belonging to the bHLH family. This protein has a basic region that binds to E-box on DNA to form a dimer or heterodimer to regulate gene expression. It was initially thought to play a key role in embryonic development. Recent evidence reveals that it is an oncogene closely related to EMT and malignant tumor growth, invasion and metastasis, and apoptosis (Ansieau et al., 2008; Zhu et al., 2016; Kim et al., 2017; Zhao et al., 2017; Georgakopoulos-Soares et al., 2020; Sonongbua et al., 2020). Twist is one of the main genes regulating EMT, and the reduction or loss of E-cadherin is the most important landmark change in EMT. Twist can negatively regulate the expression of E-cadherin, and the deletion of E-cadherin induces the expression of Twist, thus forming a positive feedback to maintain the interstitial state and induce EMT (Sasaki et al., 2009; Zhu et al., 2016; Wu et al., 2019). The Twist-related studies are focused mainly on tumors, but the regulatory mechanism of Twist in organ fibrosis, especially in DN, is not clear (Qin et al., 2012). Yang et al. (2015) established that Id2 can bind to Twist and block the expression of Collagen-I mediated by TGF-β in pulmonary fibrosis. Then, in the high-glucose stimulation of renal tubular epithelial cells, through which mechanism Twist and Id2 participate in the process of EMT still needs to be explored.

In this study, the protective effect of OMT on diabetic renal fibrosis and the putative mechanism underlying diabetic renal tubulointerstitial fibrosis were investigated *in vitro* and *in vivo*. We also assessed whether OMT can inhibit Twist-mediated tubulointerstitial fibrosis by upregulating the expression of Id2. Thus, the present study is of great clinical significance for the rational use of matrine resources and provides new experimental evidence for the treatment of DN, as well as, effective targets for the prevention and treatment of DN.

## Materials and Methods

### Experimental Animals

A total of 20 healthy 6-week-old db/db mice, SPF grade, weighting (40 ± 5) g and 10 non-transgenic db/m mice with the same background at the same age, weighing (20 ± 2) g were provided by Nanjing University-Nanjing Biomedical Research Institute (batch number: T002407, strain: BSK-DB). The study followed the guidelines of the Code of Nursing and use of Animal Science of the National Health and Medical Research Council of China. At the same time, this study is based on the animal experiment ethics committee of Guizhou Medical University (No. 1800046) for the purpose of scientific care and use of animals. The db/db mice were randomly and equally divided into the diabetic group (DM group) and the oxymatrine treatment group (OMT group). Non-transgenic age-matched db/m mice comprised the control group (NC group). After 2 weeks of adaptive feeding, the mice in the OMT group were intraperitoneally injected with OMT 120 mg/kg⋅d for 8 weeks. 24-hour urine was collected before the mice were sacrificed, and the eyeball was removed and blood was taken under ether anesthesia after 4-hour fasting. Also, the urine protein and fasting blood glucose were measured. Some kidney tissues were fixed with 4% paraformaldehyde at room temperature for paraffin sections, and the rest were stored at −80°C in the refrigerator for RNA and protein extractions.

### Double Staining of Renal Histology and Immunofluorescence

The kidney tissue was fixed with 4% neutral formaldehyde, and then the kidney tissue block was dehydrated with ethanol gradient. After embedding in paraffin, it was fixed in a microtome for continuous sectioning (thickness is 3 μm). The cut tissue slices were unfolded and fixed on the glass sheet. HE, Masson, and PAS staining was performed following the instructions of the kit. The morphological and structural changes of the kidney tissue were observed under a light microscope. Paraffin sections were taken for tissue immunofluorescence staining. First, the paraffin sections were placed in an oven at 60°C for 2 h and dehydrated by gradient ethanol. Add 1.0 mol/L pH 6.0 citrate buffer solutions and place in microwave oven to repair antigen. Cellular immunofluorescence was directly fixed with 4% paraformaldehyde and followed by the same follow-up experiment as tissue staining, that is, 30 ml/L H_2_O_2_ deionized water, 5% bovine serum albumin was sealed at room temperature for 30 min. The first antibody was incubated (rabbit anti-Twist polyclonal antibody, rabbit anti-Id2 polyclonal antibody, mouse anti-Collagen-IV monoclonal antibody (Sigma, United States), rabbit anti-Fibronectin polyclonal antibody, rabbit anti-α-SMA polyclonal antibody (Santa Cruz Biotech, Santa Cruz, CA, United States). Mouse anti-E-cadherin monoclonal antibodies (CST, United States) were diluted at 1:80 overnight. The slices incubated with phosphate buffer solution (PBS) were used as negative control. They were subjected to rewarming at room temperature for 30 min, washing three times, for 5 min each time. The corresponding CY3 labeled sheep anti-mouse IgG (1:400) or FITC-labeled sheep anti-rabbit IgG fluorescent second antibody (1:200, Santa Cruz, United States) were incubated for 1 h. After washing PBS, the nucleus was stained with DAPI solution (Sigma, United States). Finally, the film was sealed with anti-fluorescence quenchant and observed and photographed under a fluorescence microscope (Olympus FV 1000, Olympus, Japan).

### Cell Culture, Cell Administration and Cell Transfection

Rat renal tubular epithelial cell (NRK-52E) cell line was purchased from the Cell Bank of Chinese Academy of Sciences (Shanghai, China). NRK-52E was cultured in (DMEM, Gibco, United States) medium containing 10% fetal bovine serum (Hyclone, United States) at 37°C and 5% CO_2_. The cells were randomly divided into three groups: normal glucose control group (NG group, glucose medium containing 5.5 mmol/L) and normal glucose group (NC+OMT group), It contains 5.5 mmol/L glucose and 0.1 mg/mL OMT (Nanjing Guangrun Biological products Co., Ltd., batch number: GR-134-171229), high-glucose group (HG group, containing 30 mmol/L glucose), high glucose oxymatrine treatment group (DM+OMT group, It contains 30 mmol/L glucose and 0.1 mg/mL OMT). The shRNA plasmid expressing Id2 gene was transfected into NRK-52E cells, and the pCMVPuro04-Id2 plasmid was designed in Yi le Biotech (Shanghai). The structure of the plasmid was as follows: Forward pGL-F: 5′-TAATACGACTCACTATAGG-3′, Reverse pGL-R:5′-GCCGGGCCTTTCTTTATG-3′. The construction sequence of siRNA with low Id2 gene (Jima, Shanghai) was as follows: Forward: 5′-GCAGCACGUCAUCGAUUAUTT-3′, Reverse:5′-AUAAUCGAUGACGUGCUGCTT-3′. An equivalent of 4 × 10^5^ NRK-52E cells/well were inoculated into 6-well plate. At 80% confluency, the corresponding plasmids and siRNA were transfected with Lipofectamine 3000 reagent (Invitrogen, United States) for 6-hour in medium containing 1% FBS; and high glucose stimulation and drug intervention were carried out simultaneously. Other experiments were carried out after 48 h.

### Western Blot

The renal cortex homogenate and cell culture samples were dissolved in RIPA lysis buffer (Beyotime, Jiangsu, China). After centrifugation, the supernatant was removed and the protein concentration was detected according to the BCA kit instructions. The protein was isolated by polyacrylamide gel electrophoresis (proteins between 40 and 280 kDa were separated by 8% gel, proteins between 10 and 40 kDa were separated by 15% gel) after boiling with an equal volume of 5× sample buffer, and the membrane was blocked with 50 g/L skim milk. After washing the membrane with TBST, we added mouse anti-β-actin antibody (1:4000), rabbit anti-Id2 (1:1000), rabbit anti-Twist antibody (1:1000), rabbit anti-α-SMA antibody (1:1000), rabbit anti-Fibronectin antibody (1:1000), mouse anti-collagen-IV antibody (1:1000), or mouse anti-E-cadherin antibody (1:1000), followed by overnight incubation at 4°C. Further, after washing, the corresponding horseradish peroxidase labeled goat anti-mouse IgG or horseradish peroxidase labeled goat anti-rabbit second antibody IgG (1:4000, PMK, China) were incubated at room temperature for 1-h. We employed enhanced chemiluminescence, Bio-Rad gel imaging system (Bio-Rad company) exposure, and Image Lab 5.1 software processing and analysis of images.

### Real-Time PCR

Total RNA was extracted from renal tissues and cells by TRIzol reagent (Ambion). Then cDNA was synthesized by RevertAId^TM^ First Strand cDNA Synthesis Kit (Thermo, United States). Quantitative PCR was detected by 2 × SuperReal PreMix Plus (SYBR Green) (TIANGEN, Beijing, China) and iQ SYBR Green SuperMix (Bio-Rad). The primers of each gene were designed by DNA MAN software and synthesized by Generay Biotech Co., Ltd. (Shanghai, China). The gene expression was related to the expression level of β-actin, and the data were processed by 2^–ΔΔCt^ method.

### Co-immunoprecipitation

Immunoprecipitation test was carried out according to the instructions of the kit using Dynabeads Protein G Immunoprecipitation Kit (10007D, Invitrogen, United States). First, the cell lysate was added, the cell supernatant was extracted, the magnetic beads were pretreated, the 5 μL rabbit anti-Id2 antibody was incubated at room temperature for 1 h, or the 5 μL mouse anti-Twist antibody was added. In IgG group, 5 μL normal rabbit IgG antibody or normal mouse IgG antibody (PMK, China) was added to form antibody-magnetic bead complex, and then incubated at room temperature for 1 h. The antigen in the supernatant was combined with the corresponding antibody magnetic bead complex to form an antigen-antibody-magnetic bead complex. Then, we performed 1000 rpm centrifugation for 2 min, added 20 μL of 5× sample buffer at 70°C for 10 min, and conducted Western blot.

### Dual-Luciferase Reporter Assay

The ampicillin-resistant glycerol bacteria (Shanghai Leyi) containing Luciferase E-cadherin promoter sequence pGL3-rno-Cdh1 was constructed and the bacteria were amplified and the plasmid was extracted. The day before the transfection, NRK-52E cells with good logarithmic growth were digested by trypsin and inoculated with 20% to 24-well cell culture plate at 37°C for 5% CO_2_. 24-hour after cell inoculation (50–60%), the transfection operation was carried out according to the instructions of the transfection reagent. Three multiple holes were set in each group. After 5 min, the two solutions were evenly mixed, followed by incubation for 20 min at room temperature. Then, the transfection complex of 50 μL was transferred into the 24-well plate covered with cells, and 500 μL was replenished after 6-hour. Next, 48-hour after the transfection, the cells were lysed, 12,000 rpm, centrifuged for 15 min, the supernatant was taken; each pore sample was of 100 μL. The Luciferase activity was detected by Dual-Luciferase Reporter Assay System (Promega, cat: E1960), and the ratio of Renilla Luciferase reading to Firefly Luciferase reading was measured. The structure of the constructed plasmid is as follows: Forward pGL-F:5′-CTAGCAAAATAGGCTGTCC-3′, Reverse pGL-R:5′- GCCGGGCCTTTCTTTATG-3′.

### Statistical Analysis

SPSS 19.0 statistical software was used for data processing and statistical analysis. The experimental data were expressed by mean ± standard deviation (mean ± SD). First, the normality and homogeneity of variance were tested. After satisfying the homogeneity of normal distribution and variance, single factor analysis of variance was used for comparison between groups. There was significant difference between the two groups (*P* < 0.05).

## Results

### The Effect of OMT on EMT and ECM in Mouse Kidney

#### General Changes and Biochemical Indicators of the Mice in Each Group

Compared with the NC group, blood glucose (BG), 24-hour urine protein (24 h UP), serum total cholesterol (TC) and blood triglyceride (TG) in the DM group were significantly increased and statistically significant. Compared with the DM group, the 24-hour urinary protein, serum total cholesterol and blood triglyceride in the OMT group were significantly lower than those in the control group. See Table 1.

**TABLE 1:** Biochemical indexes of mice in each group (*n* = 6, $\bar{x}\pm s$).

**Specimen**	**BG/(mmol/L)**	**24 h UP/mg**	**TC/(mmol/L)**	**TG/(mmol/L)**
NC	7.530.38	7.610.42	2.210.39	1.330.30
DM	38.363.95**	36.345.24**	4.150.48*	2.590.33*
OMT	32.435.97	24.519.02^#^	2.490.76^#^	1.380.13^#^

*P < 0.05, **P < 0.01 vs. NC group; ^#^*P* < 0.05 vs. DM group.

#### Pathological Changes of Kidney Tissue in Each Group of Mice

**FIGURE 1:**
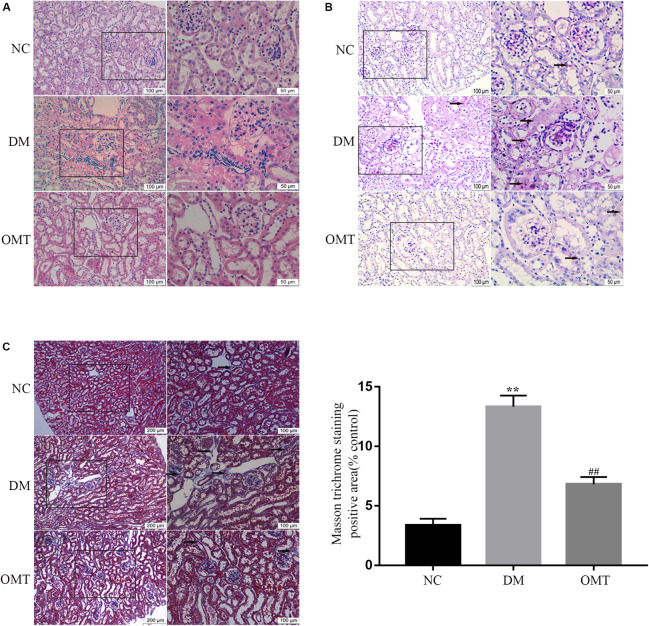
Morphological changes of renal tissue in mice in NC, DM and OMT groups. **(A)** Renal tissues of the NC, DM and OMT groups were stained with hematoxylin on the scale of 100 and 50 mm; **(B)** PAS staining of kidney tissue in NC, DM, and OMT groups was 100 and 50 mm; **(C)** Masson staining of renal tissues in NC group, DM group and OMT group; the scales were 200 and 100 mm (**P < 0.01 vs. NC group; ^##^*P* < 0.01 vs. DM group).

The results of HE staining showed that in NC group, the outline of glomerulus was clear, the epithelial cells of renal tubules were neatly arranged, with intact basement membrane, and without inflammatory cell infiltration in the stroma. In DM group, most of renal tubular epithelial cells disintegrated with vacuolar degeneration, renal tubular lumen dilated obviously and a large number of inflammatory cells infiltrated in the renal interstitial area. After OMT treatment, the glomerular and tubular lesions were improved and the infiltration of inflammatory cells in the interstitium was reduced in the OMT group (Figure 1A). The results of PAS staining showed that the structure of glomeruli and renal tubules in NC group was clear, and there was a small amount of purplish red PAS positive staining substance, and there was no obvious abnormality. In the DM group, mesangial matrix slightly proliferated and PAS positive staining increased than those in NC group. Compared with DM group, PAS positive substance in OMT group decreased significantly (Figure 1B). The results of Masson staining showed that there was no collagen deposition in glomerular basement membrane and Mesangial area and no increase in renal tubulointerstitium in NC group. In DM group, a large number of collagen fibers were deposited in the interstitial area of glomeruli and tubules, that is, blue cord-like substances in the interstitial area of glomeruli and tubules, and the blue cord-like substances in the whole visual field in OMT group were significantly lower than those in DM group (Figure 1C).

### OMT Inhibited the Process of EMT and the Deposition of ECM in Renal Tissue of Mice

**FIGURE 2:**
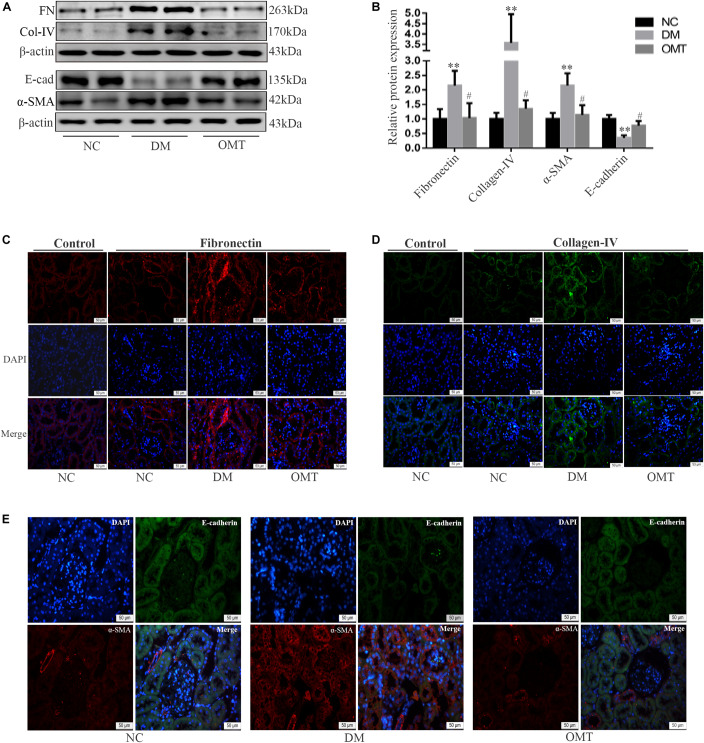
Effects of OMT on the expression of Fibronectin, collagen-IV, E-cadherin, and α-SMA in the mouse kidneys. **(A,B)** Expressions of FN (Fibronectin), E-cad (E-cadherin), α-SMA and Col-IV (Collagen-IV) in the mouse renal cortex were detected by Western blot (*n* = 6. **P < 0.01 vs. NC group; *n* = 6. ^#^*P* < 0.05 vs. DM group. **(C–E)** Immunofluorescence staining showed the expression of Fibronectin, Collagen-IV, E-cadherin and α-SMA in mouse kidney (scale is 50 μm).

The results of Western blot assay showed that compared with NC group, the expression of E-cadherin protein in DM group decreased significantly, while the expression of Twist, α-SMA, Collagen-IV and Fibronectin protein increased significantly. After the OMT treatment, the expression of E-cadherin protein significantly increased, whereas the expression of α-SMA, Collagen-IV, and Fibronectin protein significantly decreased (Figures 2A,B). The results of tissue immunofluorescence were consistent with those of Western blot assay (Figures 2C–E). We confirmed that OMT inhibited the process of EMT and the deposition of ECM in the mouse kidneys.

### By Promoting the Recovery of Id2 Expression in Mouse Renal Tissue, OMT Binds Id2 to Twist and Inhibits the Process of EMT and the Overproliferation and Abnormal Deposition of ECM

**FIGURE 3:**
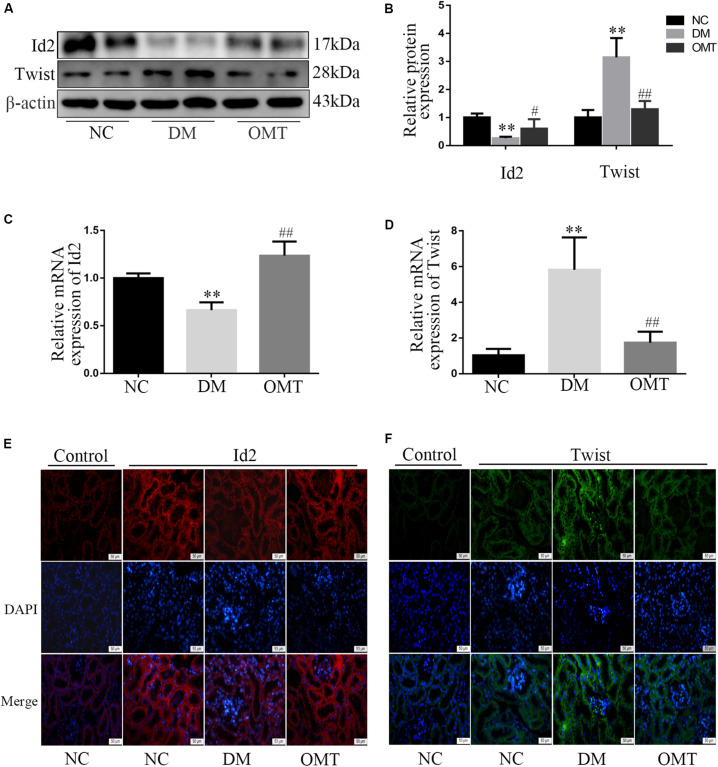
Effect of OMT on the expression of Id2 and Twist in mouse kidney tissue. **(A,B)** Western blot was used to detect the expression of Id2 and Twist protein in mouse kidney tissues (*n* = 6. **P < 0.01 vs. NC group; ^#^*P* < 0.05 vs. DM group, ^##^*P* < 0.01 vs. DM group; **(C,D)** Real-time PCR was used to detect the expression of Id2 and Twist mRNA in mouse kidney tissues (*n* = 6. **P < 0.01 vs. NC group; ^##^*P* < 0.01 vs. DM group; **(E,F)** Immunofluorescence staining showed the expression of Id2 and Twist in mouse kidney tissues (scale is 50 μm).

The results of Western blot and real-time PCR showed that the expression levels of Id2 protein and mRNA in the renal tissue of the DM group were significantly lower than those in the NC group, whereas the expression levels of Twist protein and mRNA were significantly lower. After OMT treatment, the expression levels of Id2 protein and mRNA were significantly increased, whereas the expression levels of Twist protein and mRNA were significantly decreased (Figures 3A–D). The results of tissue immunofluorescence were consistent with those of Western blot assay (Figures 3E,F).

### OMT Inhibits EMT Processes and Deposition of ECM in High-Glucose-Cultured NRK-52E Cells

**FIGURE 4:**
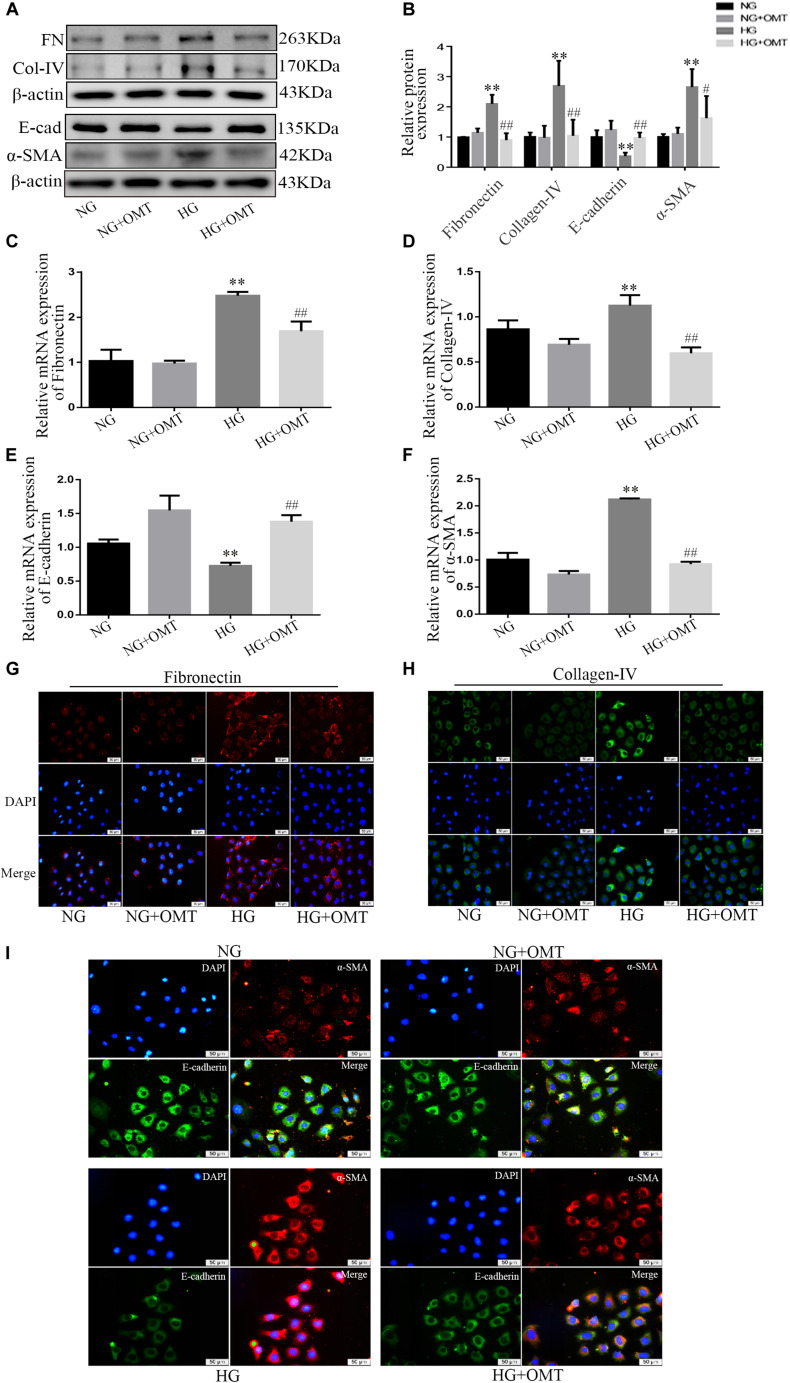
Effects of OMT on the expression of Fibronectin, Collagen-IV, E-cadherin and α-SMA in NRK-52E cells. **(A–F)** Western blot and RT-PCR were used to detect the expression of FN (Fibronectin), E-cad (E-cadherin), α-SMA and Col-IV (Collagen-IV) in NRK-52E (*n* = 3. **P < 0.01 vs. NG group; ^#^*P* < 0.05, ^##^*P* < 0.01 vs. HG group); **(G–I)** Immunofluorescence staining showed expression of Fibronectin, Collagen-IV, E-cadherin, and α-SMA in NRK-52E cells (scale bar is 50 μm).

NRK-52E cells were cultured with normal or high glucose content, and added to the samples of the NG+OMT and HG+OMT groups. The results of Western blot and real-time PCR showed that the levels of E-cadherin protein and mRNA in HG group were significantly lower than those in NG group. However, the expressions of α-SMA, Collagen-IV, Fibronectin protein and mRNA were significantly increased. Compared to the HG group, the levels of E-cadherin protein and mRNA in HG+OMT group were significantly increased, while the expressions of α-SMA, Collagen-IV, Fibronectin protein and mRNA were significantly decreased (Figures 4A–F). The results of cellular immunofluorescence showed that the fluorescence brightness of α-SMA, Collagen- IV and Fibronectin in the HG group was significantly higher than that in the NG group, mainly in cytoplasm, but not in nucleus, and the fluorescence brightness of E-cadherin in HG group was considerably darker than that in the NG group. The fluorescence brightness of α-SMA, Collagen-IV, and Fibronectin in the HG+OMT group was significantly lower than those in the HG group, whereas the fluorescence brightness of E-cadherin significantly increased (Figures 4G–I).

### In NRK-52E Cells, OMT Can Inhibit the Process of EMT and the Overproliferation and Abnormal Deposition of ECM by Promoting the Recovery of Id2

**FIGURE 5:**
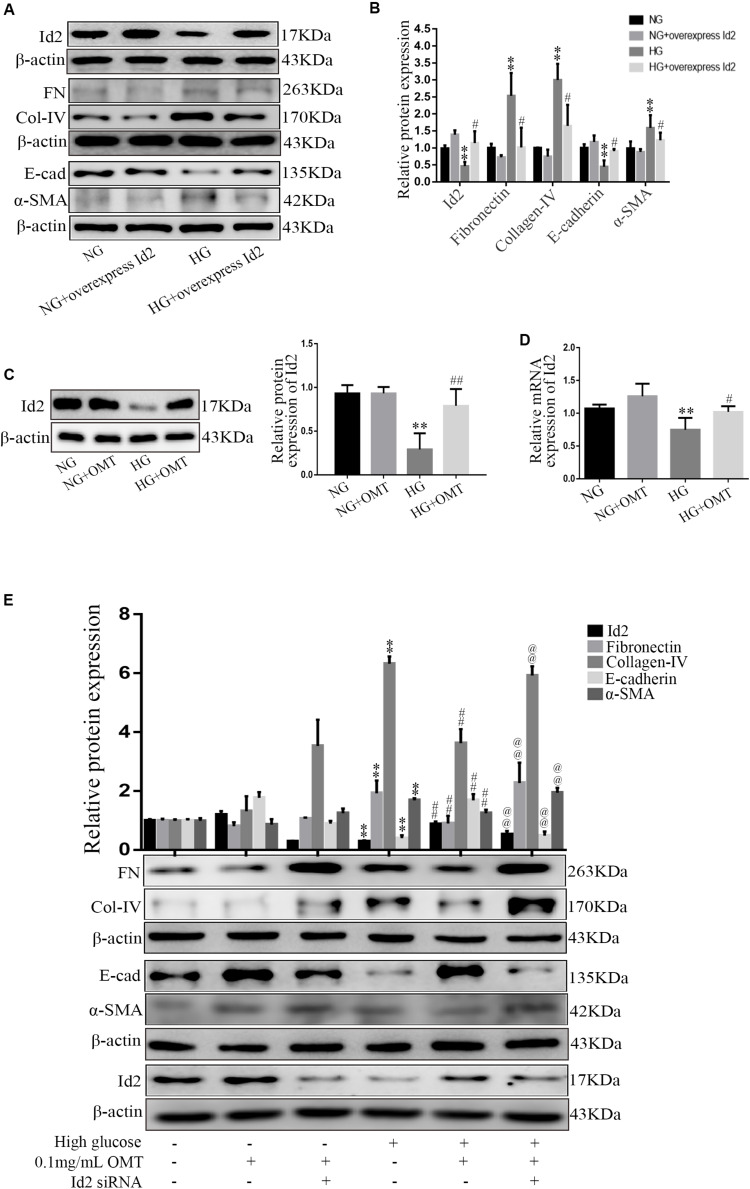
Effects of Id2 on the expression of Fibronectin, Collagen-IV, E-cadherin, and α-SMA in NRK-52E cells were observed by Western blot and Real-time PCR. **(A,B)** Western blot was used to detect the changes of FN (Fibronectin), E-cad (E-cadherin), α-SMA, and Col-IV (Collagen-IV) after Id2 expression (*n* = 3, **P < 0.01, vs. NG group; ^#^*P* < 0.05 vs. HG group). **(C,D)** Western blot and RT-PCR were used to detect the changes of protein and mRNA levels of Id2 after OMT treatment (*n* = 3, **P < 0.01 vs. NG group; ^#^*P* < 0.05, ^##^*P* < 0.01 vs. HG group); **(E)** The expression of Id2, Fibronectin, E-cadherin, α-SMA and Collagen-IV in NRK-52E was observed by Western blot and RT-PCR after OMT treatment and knock-down of Id2 after OMT treatment (*n* = 3, **P < 0.01 vs. NG group; ^##^*P* < 0. 01 vs. HG group; ^@@^*P* < 0.01 vs. HG+OMT group).

In normal glucose or high-glucose-cultured NRK-52E cells, Western blot in the NG+overexpression Id2 group and HG+overexpression Id2 group showed that the level of E-cadherin protein in HG group was significantly lower than that in NG group. The expression of SMA, Collagen-IV, and Fibronectin proteins was significantly increased. Compared to the HG group, the level of E-cadherin protein in HG+overexpression Id2 group increased significantly, while the expression of α-SMA, Collagen-IV, and Fibronectin protein decreased significantly (Figures 5A,B). NRK-52E cells cultured with normal glucose and high glucose were applied to the NG+OMT and HG+OMT groups. Using Western blot and real-time PCR, we found that the levels of Id2 protein and mRNA in HG group were significantly lower than those in NG group, while the levels of Id2 protein and mRNA in the HG+OMT group were significantly higher than those in the HG group (Figures 5C,D). The NRK-52E cells cultured with normal glucose and high glucose were treated with drugs and Id2 siRNA groups, that is, NG+OMT group, NG+OMT+Id2 siRNA group, HG+OMT group and HG+OMT+Id2 siRNA group. The results of Western blot showed that compared with NG group, the levels of Id2 and E-cadherin protein in HG group decreased significantly, while the expression of α-SMA, Collagen-IV and Fibronectin protein increased significantly. The level of E-cadherin protein in the HG+OMT group was significantly higher, whereas the expression of α-SMA, Collagen-IV, and Fibronectin protein was significantly lower in the Id2 group than in the HG groups. In the NG+OMT+Id2 siRNA and HG+OMT+Id2 siRNA groups, the levels of Id2 and E-cadherin proteins decreased significantly compared with the corresponding NG+OMT and HG+OMT groups, while that of α-SMA, Collagen-IV, and Fibronectin proteins increased significantly (Figure 5E).

### In NRK-52E Cells, OMT Inhibits the Process of EMT and ECM Deposition by Promoting the Recovery of Id2 Expression and Affecting the Transcriptional Activation of Downstream Target Genes After Id2 Binding to Twist

**FIGURE 6:**
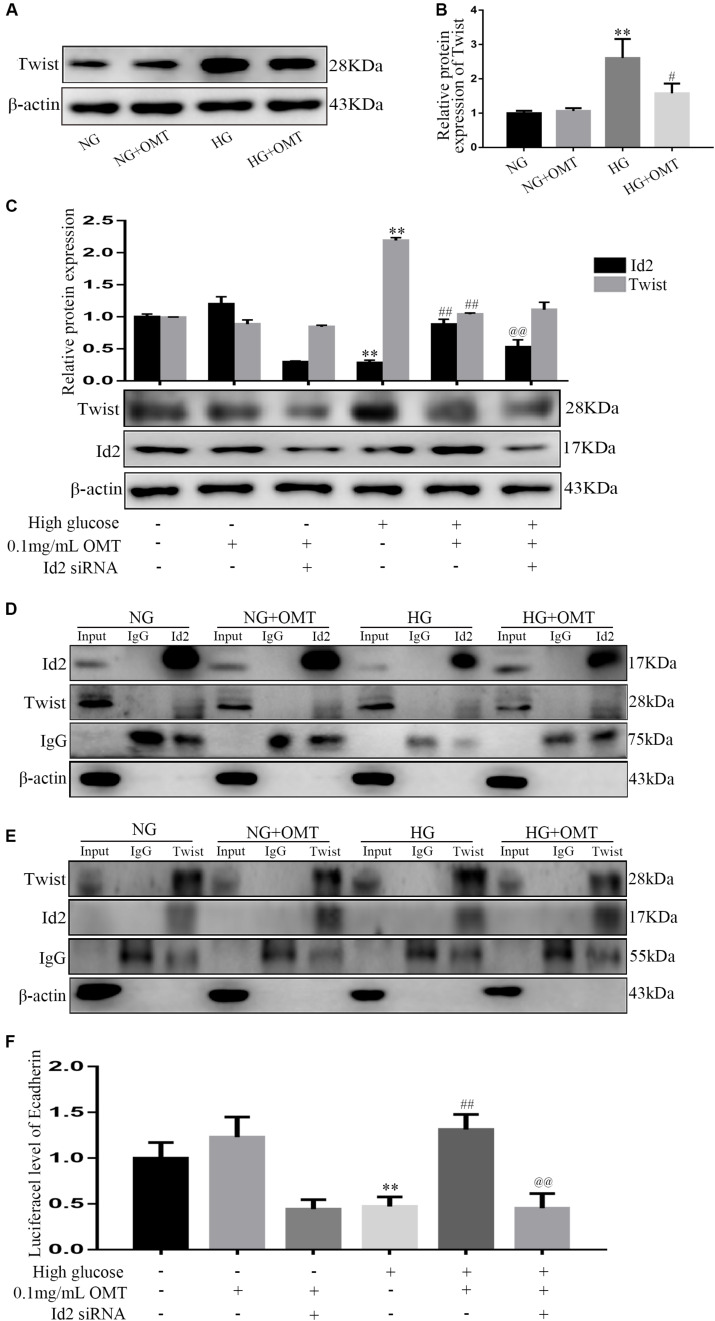
Relationship between Id2 and Twist in NRK-52E cells, established by Western blot, Co-IP, and Luciferase assays. **(A,B)** Western blot was used to detect the changes of Twist protein level after administration of OMT (*n* = 3, **P < 0.01 vs. NG group; ^#^*P* < 0.05 vs. HG group); **(C)** The protein levels of Id2 and Twist were detected by Western blot after knocking down Id2 after OMT treatment. **P < 0.01 vs. NG group; ^##^*P* < 0.01 vs. HG group; ^@@^*P* < 0.01 vs. HG+OMT group; **(D,E)** Correlation between Id2 and Twist was detected by Co-IP. **(F)** The luciferase activity of the downstream target gene of Twist, namely the promoter of E-cadherin gene, was observed by Luciferase assay after treatment with OMT and treatment with OMT to knock-down Id2. **P < 0.01 vs. NG group; ^##^*P* < 0.01 vs. HG group; ^@@^P*P* < 0.01 vs. HG+OMT group.

NG+OMT and HG+OMT groups were set up in both normal glucose and high glucose culture NRK-52E cells. The results of the Western blot revealed that the level of Twist protein in HG group was significantly higher than that in NG group, and compared with HG group. The level of Twist protein in HG+OMT group was significantly decreased (Figures 6A,B). NRK-52E cells were cultured in normal sugar and high glucose, treated with OMT and transfected with Id2 siRNA, i.e., NG+OMT group, NG+OMT+Id2 siRNA group, HG+OMT group, and HG+OMT+Id2 siRNA group. The results of Western blot showed that compared to the NG group, the level of Id2 protein in the HG group was decreased significantly, while the expression of Twist protein was increased significantly. Compared to the HG group, the level of Id2 protein in the HG+OMT group was increased significantly, and that of the Twist protein was reduced significantly. Furthermore, Compared to the HG+OMT group, the Id2 protein level was decreased significantly in the HG+OMT+Id2 siRNA group, while the expression of Twist protein was only slightly increased (Figure 6C). Immunoprecipitation assay revealed that Twist protein was a component of the complex precipitated by Id2 antibody in the NG+OMT group of NG group or in the HG+OMT group of HG group. Also, the Id2 protein was detected in the complex precipitated by Twist antibody, thereby indicating an interaction between Id2 and Twist proteins (Figures 6D,E). Then, the Luciferase assay showed that the activity of the E-cadherin promoter Luciferase was significantly reduced in the HG group as compared to the NG group. Compared to the HG group, OMT intervention promoted the recovery of the Luciferase activity of the E-cadherin promoter, while the Luciferase activity of E-cadherin promoter after transfection of Id2 siRNA was significantly lower than that of OMT intervention group stimulated by high glucose. In addition, it was confirmed that OMT inhibited the process of EMT and the deposition of ECM by promoting the restoration of Id2, which was formed by binding Id2 to Twist to form a heterodimer, thereby affecting the regulation of downstream genes by Twist (Figure 6F).

## Discussion

In recent years, the incidence of diabetes has been steadily increasing, seriously affecting human physical and mental health. Type 2 diabetes is most prevalent. Currently, the research and development of drugs for treatment of type 2 diabetes has intensified considerably spot (Santulli, 2019). In this study, we carried out both *in vivo* and *in vitro* experiments. In our *in vivo* experiments, we selected an animal model of type 2 diabetes, db/db mice, a spontaneous type 2 diabetic mouse caused by a defect in the Leptin receptor gene on chromosome 4 discovered by the Jackson Laboratory in the United States in 1966. Bulimia and obesity appeared from the age of 4 weeks, and then showed obvious characteristics of hyperglycemia, hyperlipidemia and insulin resistance with the increase of age. The pathogenesis of hyperglycemia was very similar to that of patients with type 2 diabetes mellitus. It is also an ideal animal model for the experimental study of type 2 DN in the world (Lutz and Woods, 2012; Zar Kalai et al., 2014; Zhu et al., 2019).

Our previous study found that oxymatrine played an anti-fibrotic effect in renal tubular epithelial cells (Liu et al., 2016). This experiment was confirmed again *in vivo*, and it was found that after intraperitoneal injection of oxymatrine in db/db mice for 8 weeks, The state of the mice was improved, the biochemical indexes such as fasting blood glucose, serum cholesterol and blood triglyceride were improved, the levels of Fibronectin, Collagen-IV, α-SMA protein and mRNA were decreased, while the levels of E-cadherin were increased. It is confirmed that oxymatrine can improve the degree of renal fibrosis in diabetic mice, but the specific mechanism is not very clear. Earlier studies showed that the expression of Id2 protein is closely related to fibrosis in mouse proximal renal tubular cells. With the development of EMT, the expression of Id2 decreased significantly (Gervasi et al., 2012; Vigolo et al., 2019). Our results also found that compared with the wild-type mice, the expression of Id2 in renal tissue of db/db mice decreased significantly, and the level of Twist increased significantly. After treatment with oxymatrine, the expression of Id2 recovered and Twist decreased. Therefore, we speculated that Id2 and Twist were key regulatory factors of diabetic renal fibrosis EMT, and there may be a regulatory relationship between them. Nevertheless, it was still unclear what the exact relationship between Id2 and Twist in diabetic renal fibrosis was and whether oxymatrine exerted protective effects in diabetic nephropathy through both of them. To address these questions, we performed the present study.

**FIGURE 7:**
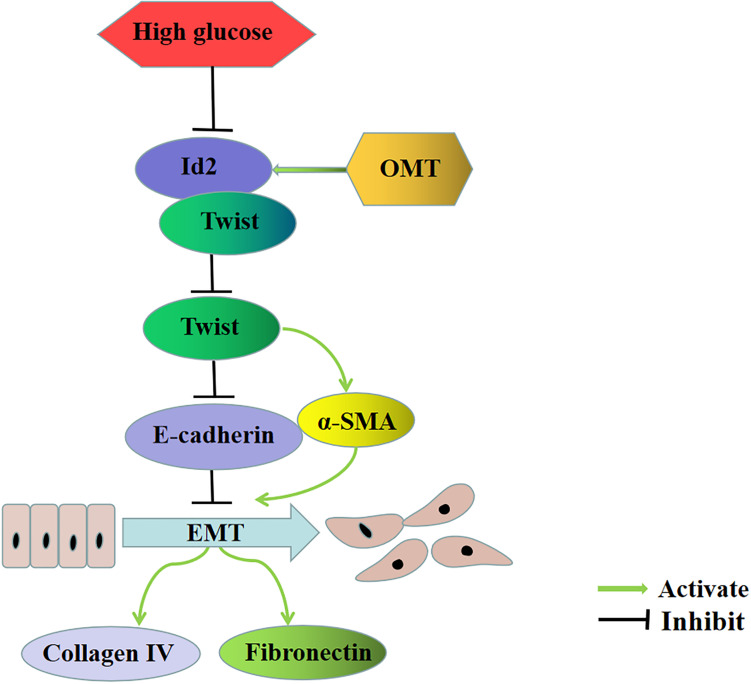
Oxymatrine is involved in the main signal pathway of diabetic renal tubulointerstitial fibrosis. OMT can block the inhibitory effect of HG on Id2, promote the recovery of Id2, and further promote the binding of Id2 and Twist, thus affecting the regulation of downstream target genes E-cadherin and α-SMA by Twist, inhibiting the process of EMT, and downregulating the expression of ECM such as Collagen-IV and Fibronectin.

Firstly, we stimulated the renal tubular epithelial cells of rats with high glucose and treated with oxymatrine to explore the effects of OMT on EMT and ECM. The results showed that the EMT process of NRK-52E cells cultured with high glucose was reversed and the secretion of ECM was decreased after treatment with oxymatrine, suggesting that OMT can reverse the process of EMT and reduce the deposition of ECM *in vitro* and *in vivo*. Second, through the transfection of Id2 plasmid, the process of EMT and the deposition of ECM were improved. Compared with the treatment group treated with OMT, no differences were found in the effect exerted. It is suggested that the anti-EMT and ECM effect of OMT may play a role by promoting the recovery of Id2 expression. Therefore, after the administration of OMT, we knocked down the Id2, and found that the anti-EMT and ECM deposition effect of OMT was significantly diminished, which confirmed that OMT could play the role of anti-diabetic renal fibrosis by promoting the recovery of Id2 expression. Finally, in the course of the experiment, we found that the expression of Twist was significantly lower after the OMT treatment and that Id2 was a transcription factor of the HLH family. Id2 protein lacked the basic region bound to DNA and needed to bind to bHLH to form a heterodimer. Twist is a member of the bHLH family (Yang et al., 2015; Li N. et al., 2019). Thus, we speculated that oxymatrine promoted the recovery of Id2, which inhibited Twist itself or affected the gene regulation of Twist, and play an anti-diabetic role in renal fibrosis. Therefore, by knocking down Id2, while giving OMT, we found that after knocking down Id2, Twist itself has not changed significantly, suggesting that OMT may affect the transcriptional activation of downstream target genes through the binding of Id2 and Twist to affect the EMT process. Hence, we conducted a Co-IP experiment on Id2 and Twist protein and established that no matter whether it was normal, high glucose or medication group, The Twist protein was found in the complex precipitated by the Id2 antibody. Also, the Id2 protein was detected in the complex precipitated by the Twist antibody, indicating a mutual binding between Id2 and Twist proteins. Then the Luciferase reporter gene experiment was carried out by constructing the downstream target gene of Twist, that is, the promoter of E-cadherin gene. It was found that the Luciferase activity of E-cadherin promoter was significantly lowered under high-glucose culture, and recovered after oxymatrine treatment. After the treatment with oxymatrine and knocking down Id2, the luciferase activity of E-cadherin promoter decreased significantly, which confirmed once again that Id2 did not affect the expression of Twist itself, but through the effect of binding to Twist. Thus affecting the expression of downstream target genes to play the role of EMT and ECM deposition, indicating that oxymatrine can promote the recovery of Id2 and further promote the binding of Id2 and Twist, thus inhibiting the regulation of downstream target genes by Twist and exerting anti-diabetic functions against renal fibrosis (Figure 7).

In this study, we investigated the protective mechanism of oxymatrine on renal tubular epithelial cell injury, induced by high glucose, and confirmed the interaction between Id2 and Twist. Our findings provide a new experimental basis for the rational utilization of *Sophora flavescens* resources, elucidating its target and increasing the function of “diabetic nephropathy,” and brings new hope for delaying the progress and treatment of diabetic renal fibrosis.

## Data Availability Statement

The raw data supporting the conclusions of this article will be made available by the authors, without undue reservation, to any qualified researcher.

## Ethics Statement

This study follows the guidelines of the National Animal Health Care and Use Guidelines of the National Health and Medical Research Council of China, and the protocol has been approved by the Guizhou Medical University the Animal Care Welfare Committee.

## Author Contributions

YiX and CP participated in the design and conducted the experiment, analyzed the results, and wrote the manuscript. YaX, DL, ZY, and ZL contributed to the conduction of the experiments and the maintenance of cell culture. MS, YW, and FZ reviewed the results and manuscript. BG designed and supervised the project and reviewed the results and manuscript.

## Conflict of Interest

The authors declare that the research was conducted in the absence of any commercial or financial relationships that could be construed as a potential conflict of interest.
